# Clinical outcomes of radiation therapy for clinical T4b oesophageal cancer with airway invasion

**DOI:** 10.1186/s13014-018-1196-6

**Published:** 2018-12-14

**Authors:** Hakyoung Kim, Dongryul Oh, Yong Chan Ahn, Keunchil Park, Myung-Ju Ahn, Se-Hoon Lee, Jong-Mu Sun, Young Mog Shim, Jae Ill Zo, Yong Soo Choi, Hong Kwan Kim, Jong Ho Cho

**Affiliations:** 1 Department of Radiation Oncology, Samsung Medical Center, Sungkyunkwan University School of Medicine, Seoul, Republic of Korea; 2 Department of Medicine (Hemato-oncology), Samsung Medical Center, Sungkyunkwan University School of Medicine, Seoul, Republic of Korea; 3 Department of Health Sciences and Technology, Samsung Advanced Institute of Health Science and Technology, Sungkyunkwan University, Seoul, Republic of Korea; 4 Department of Thoracic Surgery, Samsung Medical Center, Sungkyunkwan University School of Medicine, Seoul, Republic of Korea

**Keywords:** T4b oesophageal cancer, Radiation therapy, Oesophageal fistula

## Abstract

**Background:**

Oesophageal cancer with airway invasion presents a challenge for therapy and often has serious complications. We analysed the clinical outcomes of radiation therapy (RT) in patients with clinical T4b oesophageal cancer with airway invasion.

**Methods:**

We retrospectively reviewed the medical records of 73 patients with oesophageal cancer who had clinical T4 disease and who received RT between January 1994 and June 2017. Among them, 47 patients with clinical T4b disease with airway invasion were included in this study; 31 had gross invasion on bronchoscopy and 16 had extrinsic compression with mucosal change. We investigated the survival outcomes, clinical courses, and toxicities.

**Results:**

The median survival (MS) time was 9 months. The 1- and 2-year overall survival (OS) rates were 41.4 and 27.4%, respectively. The MS times for patients treated with curative or palliative aims were 15 and 4 months, respectively (*p* = 0.001). Seven patients (14.9%) had fistulae at diagnosis; after RT, three had no change in size, three closed, and one had increased. Newly developed oesophageal fistulae after treatment were observed in 13 patients (27.7%). The median time to a newly developed fistula was 3 months (range, 1–15). Among them, a fistula was closed in only one patient. Death from aspiration pneumonia occurred in one patient who had a fistula at diagnosis and in nine patients who newly developed fistulae after treatment. Severe oesophageal bleeding causing death occurred in two patients. Patients with gross invasion on bronchoscopy had a higher risk of developing a fistula than did patients with mucosal change (37.5% vs. 25.0%, respectively).

**Conclusions:**

Even for clinical T4b disease with airway invasion, RT with a curative aim showed acceptable survival outcomes in patients with good performance status and no distant metastasis at initial diagnosis. However, the risk of fistula development associated with fatal events remains high. Further study is warranted to decrease the risks of treatment and improve clinical outcomes.

**Trial registration:**

Retrospectively registered.

## Background

In patients with oesophageal cancer, airway invasion with or without a fistula was considered a contraindication for radiation therapy (RT) and associated with a dismal prognosis. Left untreated, patients developed pulmonary infections and sepsis with median survivals of only 1 to 6 weeks [1, 2]. However, RT significantly improved the survival rate compared to that for only supportive care [2, 3]. Additionally, closures of fistulae and occasional long-term survivors were reported. Several studies reported a significant improvement in local control and overall survival with concurrent chemoradiotherapy (CCRT) compared with RT alone, even though it was associated with severe complications [4–9]. In the current National Comprehensive Cancer Network (NCCN) guidelines, definitive CCRT is recommended for the treatment of patients with clinical T4b oesophageal cancer, and chemotherapy alone can be considered in cases of tracheal invasion. RT can alleviate symptoms and increase the chance of cure for oesophageal cancer patients with airway invasion with or without a fistula, but the treatment can have severe complications. In this study, we reviewed the clinical course of cT4b oesophageal cancer patients with airway invasion, who received RT.

## Methods

### Patients

After receiving approval from the Institutional Review Board (IRB), we retrospectively reviewed the medical records of 73 patients with oesophageal cancer who had clinical T4 disease and who received RT between January 1994 and June 2017. Among them, 47 patients with clinical T4b disease and airway invasion were included. Patients with (1) clinical T4a disease invading the pleura, pericardium, or diaphragm (*N* = 16), (2) clinical T4b disease without bronchoscopy (*N* = 7), (3) clinical T4b disease with other structural involvement (*N* = 2), and (4) follow-up loss less than 1 month after the end of RT (*N* = 1) were excluded.

### Diagnostic and staging scheme

All tumours were staged based on the seventh edition of the American Joint Committee on Cancer (AJCC) criteria. The tumour assessment consisted of complete history-taking, physical examination, complete blood counts, chemistry profiles, chest radiography, esophagogastroduodenoscopy (EGD) with biopsy, and a computed tomography (CT) scan of the chest and upper abdomen. Bronchoscopy with or without biopsy was performed when tumour invasion into the wall of the trachea and/or bronchus was suspected. Whole-body ^18^F-fluorodeoxyglucose positron-emission tomography with CT (FDG-PET-CT) scans were performed for diagnosis and staging.

### Treatment

All patients underwent contrast-enhanced CT for RT simulation. Primary tumours and metastatic lymph nodes were delineated as the gross tumour volume (GTV) based on imaging and endoscopic findings. The clinical target volume (CTV) of the primary tumour included the primary GTV plus 2- to 3-cm margins in the craniocaudal directions and a 0.5-cm margin in the circumference. The nodal CTV was delineated by placing a 1-cm margin in all directions from the nodal GTV. The planning target volume (PTV) was defined as a 0.5- to 0.7-cm margin in all directions from the CTV to account for respiratory motion and daily setup errors. The planned total dose depended on the therapeutic aim, as described in Table 1. Nearly all patients received three-dimensional conformal RT (3D-CRT), typically through three or four coplanar fields using 4-, 6-, or 10-MV photons from a linear accelerator. Intensity-modulated radiation therapy (IMRT) was widely used toward the end of 2016 to treat oesophageal cancer in our institution and was administered to nine patients (19.1%) in this study who were treated with a curative aim. Two cycles of intravenous chemotherapy (5-fluorouracil [5-FU] 1000 mg/m^2^/day for 4 consecutive days plus cisplatin 60 mg/m^2^/day on the first day) were administered at 3-week intervals for definitive CCRT.

**Table 1:** Patient and tumor characteristics (*n* = 47)

Characteristics	Number of patients	%
Age [years; median (range)]	62 (44–84)	
Gender		
Male	45	95.7
Female	2	4.3
ECOG performance status		
1	33	70.2
2	13	27.7
3	1	2.1
Tumor site		
Cervical	5	10.6
Upper thoracic	18	38.3
Middle thoracic	24	51.1
Clinical T4b category^a^		
Gross invasion in bronchoscopy	31	66.0
Mucosal change in bronchoscopy	16	34.0
Stage (AJCC 7th)		
IIIC	41	87.2
IV	6	12.8
Treatment		
Definitive CCRT	20	42.5
Definitive RT alone	7	15.0
Induction CT followed by RT	4	8.5
Neoadjuvant CCRT	8	17.0
Palliative RT	8	17.0
Radiotherapy dose		
−39 Gy	10	21.3
40–49 Gy	9	19.1
50–59 Gy	5	10.6
60–70 Gy	23	49.0
Radiotherapy technique		
3DCRT	38	80.9
IMRT	9	19.1

*Abbreviation: ECOG* Eastern Cooperative Oncology Group, *AJCC* American Joint Committee on Cancer, *CCRT* Concurrent chemoradiotherapy, *RT* radiotherapy, *CT* chemotherapy, *3DCRT* 3-dimensional conformal radiation therapy, *IMRT* Intensity-modulated radiotherapy

^a^Suspicious airway invasion on computed tomography image

### Surveillance

The patients were evaluated at 1 month after RT, then were asked to visit every 3 to 4 months for 2 years and every 6 months thereafter to detect disease progression during follow-up. EGD or imaging study with either chest CT or PET-CT scans was performed on each visit. The Revised Response Evaluation Criteria in Solid Tumors (RECIST) guidelines (Version 1.1) were used for tumour response evaluation. The toxicities were evaluated according to the criteria of the Common Terminology Criteria for Adverse Events (CTCAE v.4.03).

### Statistical analysis

Overall survival (OS) was defined as the time from the start date of the initial treatment until the date of death from any cause or until the latest documented follow-up. The survival rates were estimated using the Kaplan–Meier method and were compared using log-rank tests for categorical variables. To compare the incidence of fistula development according to the bronchoscopic findings, chi-squared or Fisher’s exact tests were used. *P* < 0.05 was considered statistically significant in two-tailed tests. Statistical analysis was performed using IBM SPSS Statistics for Window, version 24.0 (IBM Corp., Armonk, NY, USA).

## Results

### Patient and tumour characteristics

**Fig. 1:**
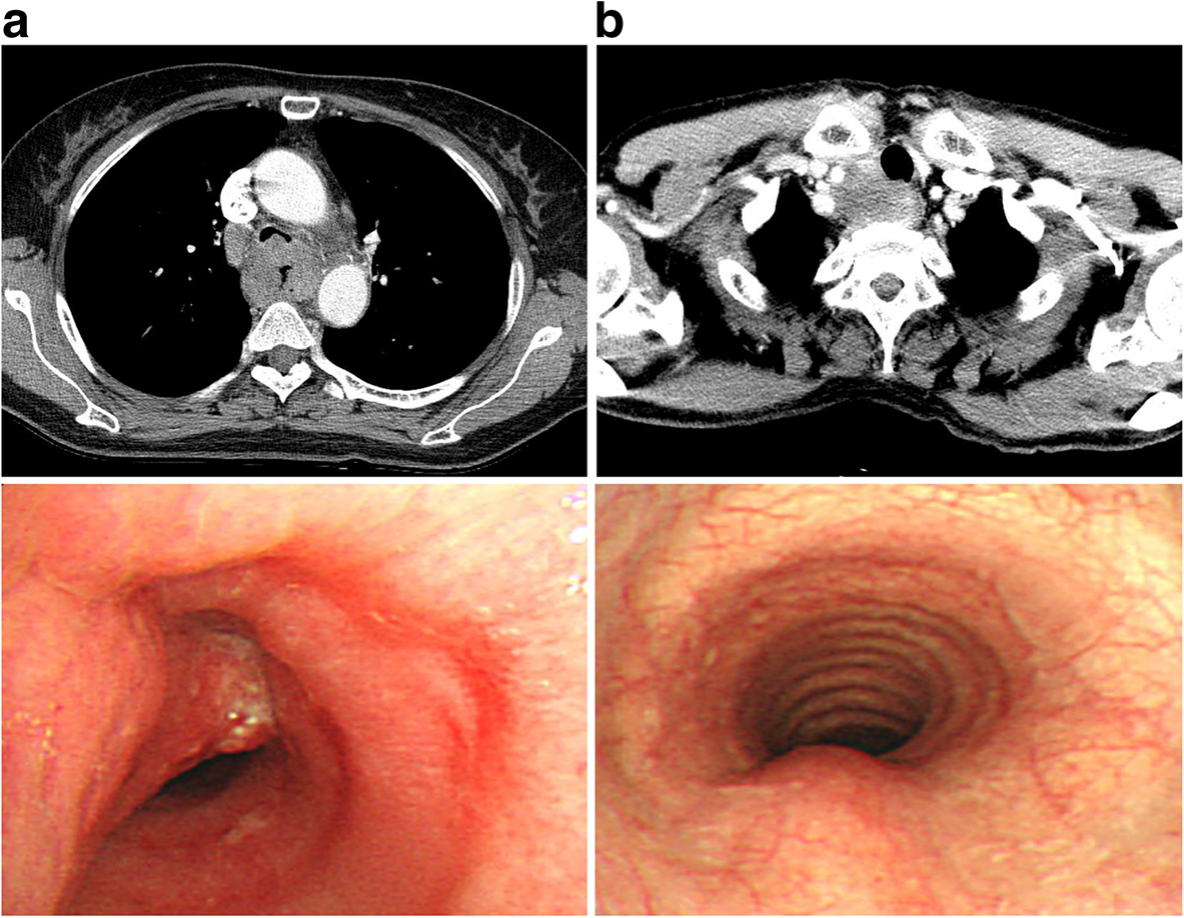
Chest CT and bronchoscopy images in patients with gross invasion (**a**) and mucosal change (**b**) on bronchoscopy

The overall patient and tumour characteristics are described in Table 1. The median age of the population was 62 years (range, 44 to 84 years). A large proportion of the population was male (95.7%). Most of the tumours were located in the upper or middle thorax (38.3 and 51.1%, respectively) and all showed squamous-cell carcinoma in the pathologic report. Among the 47 patients with clinical T4b disease and airway invasion on chest CT, gross invasion was confirmed by bronchoscopy in 31 (66.0%, Fig. 1a) and mucosal change was observed in 16 (34.0%, Fig. 1b). Most of the patients were treated with a curative aim (78.7%), except for 10 patients treated with a palliative aim because of either distant metastasis at initial diagnosis (six patients) or poor performance status (four patients). Among the patients treated with neoadjuvant CCRT, all underwent operation except for one patient who had a cardiac event during RT and stopped therapy at 20 Gy/10Fx.

### Survival outcomes

**Fig. 2:**
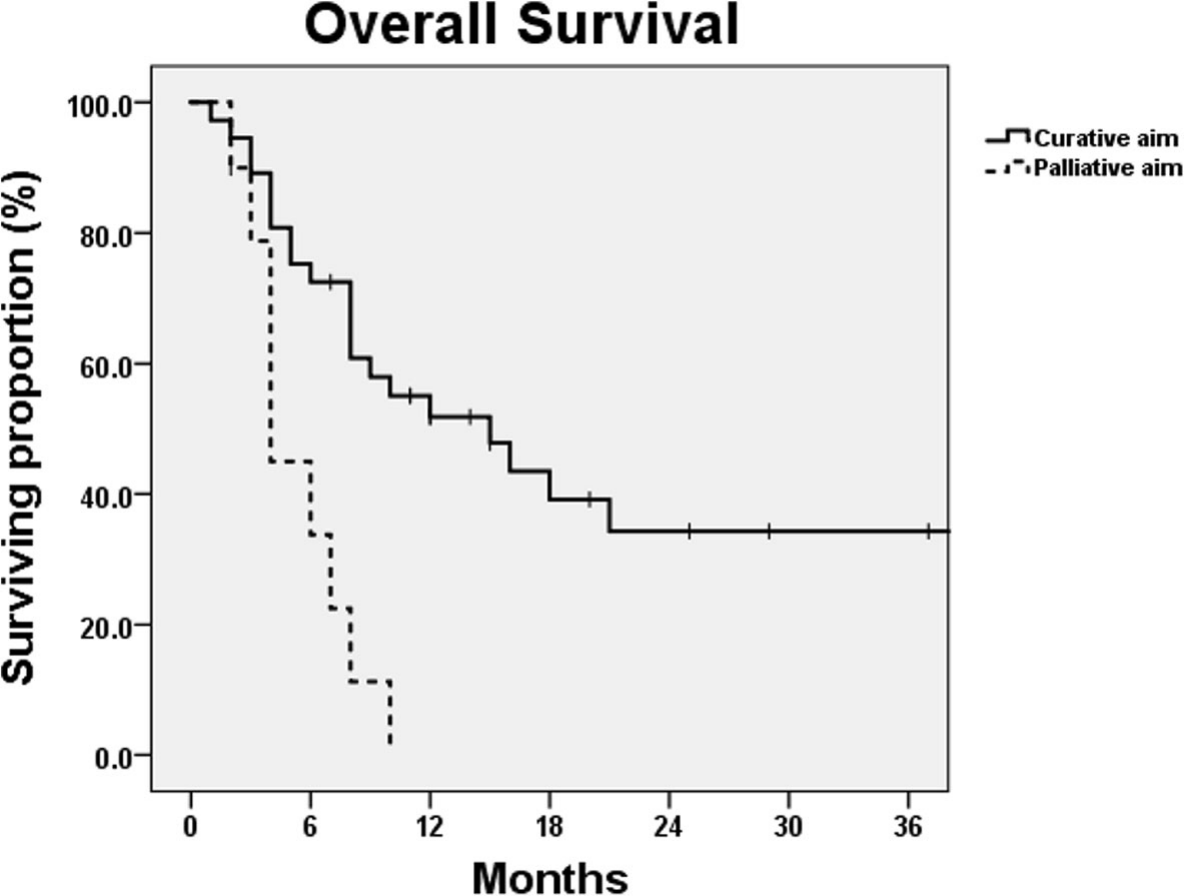
Overall survival curves after treatment according to the treatment aim. The median survival times for patients treated with curative and palliative aims were 15 and 4 months, respectively (*p*-value = 0.001)

**Fig. 3:**
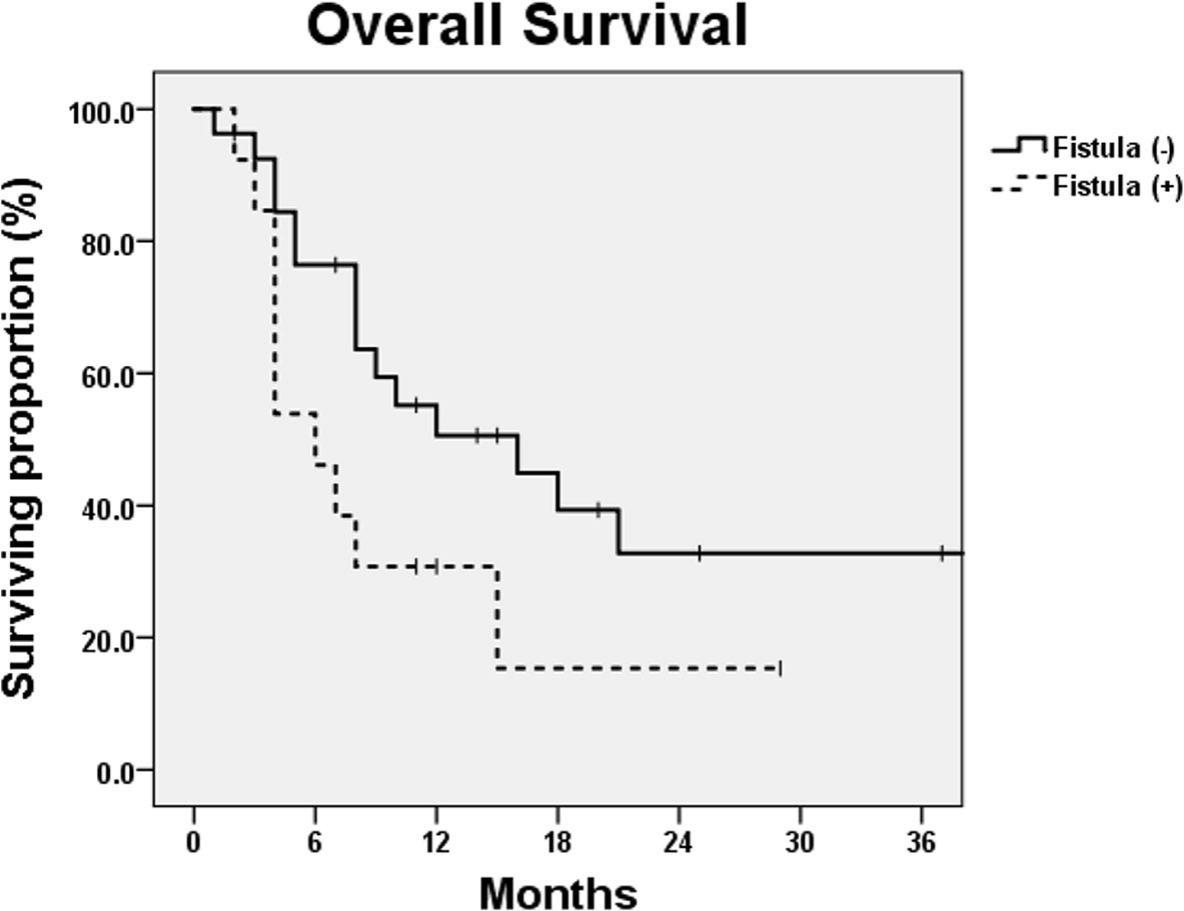
Overall survival curves after treatment according to fistula development. The median survival times for patients with no fistula after treatment and who developed a fistula were 16 and 6 months, respectively (*p*-value = 0.071)

The median follow-up duration was 8 months (range, 1 to 93 months). The median survival (MS) time was 9 months. The 1- and 2-year OS rates were 41.4 and 27.4%, respectively. The MS times for patients treated with curative or palliative aims were 15 and 4 months, respectively (*p* = 0.001) (Fig. 2). For cases with a curative aim, the 1- and 2-year OS rates were 51.8 and 34.3%, respectively. The MS times for patients treated with definitive CCRT, neoadjuvant CCRT, and definitive RT alone were 18, 15, and 6 months, respectively (*p* = 0.009). For induction chemotherapy, the estimated MS time was not reached. The presence of a fistula at initial diagnosis was not associated with decreased OS (*p* = 0.929). Among patients without fistula at initial diagnosis, those who developed a fistula after treatment showed inferior OS, although the difference was not statistically significant (*p* = 0.071). The MS times for patients without fistula after treatment and those who developed a fistula were 16 and 6 months, respectively. Their 1-year OS rates were 50.6 and 30.8%, respectively (Fig. 3).

### Clinical courses and toxicities

Among the 47 patients with clinical T4b disease and airway invasion, seven (14.9%) had a fistula at diagnosis. Although fistula formation was observed, none of the patients had a fever or associated pneumonia. All patients reported severe dysphagia caused by the primary tumour. The clinical courses of patients with a malignant oesophageal fistula at initial diagnosis are summarized in Table 2. Definitive RT or CCRT with a planned total dose of 60 Gy or greater was delivered to five patients, except for two patients who received palliative RT because of poor performance status. After RT, three patients had no change in fistula size, three fistulae had closed, and one fistula was aggravated. Two of the three patients who showed a closing of their fistula could eat a normal regular diet at the last follow-up date. One patient who remained on tube feeding and showed closing of the fistula at the last visit died from an unknown cause.

**Table 2:** Clinical courses of patients with malignant esophageal fistula at initial diagnosis

No.	Age/Sex	PS	Site	Treatment	RT technique	Change of fistula	Diet at last f/u	Survival (month)	Status
1	53/M	2	BEF	Palliative RT, 30Gy/10Fx	3DCRT	Closed at 2 months after RT	Tube feeding	2	Dead of unknown cause
2	56/F	1	TEF	Definitive CCRT with FP, 66Gy/33Fx	3DCRT	Closed^a^	Oral intake	93	Alive with no evidence of disease^a^
3	68/M	1	TEF	Definitive RT alone, 60Gy/20Fx	3DCRT	Closed at 2 months after RT	Oral intake	6	Dead of disease
4	62/M	1	TEF	Definitive CCRT with FP, 70Gy/35Fx	3DCRT	No change	Tube feeding	8	Dead of unknown cause
5	58/M	2	TEF	Palliative RT, 45Gy/18Fx	3DCRT	Aggravated	Tube feeding	10	Dead of disease (aspiration pneumonia)
6	60/M	1	BEF	Definitive RT alone, 22Gy/11Fx (incomplete)	3DCRT	No change	Tube feeding	3	Dead of disease
7	54/M	1	T-BEF	Definitive CCRT with FP, 60Gy/30Fx	IMRT	No change	Tube feeding	12	Alive with disease

*Abbreviation: PS* performance status, *RT* radiotherapy, *BEF* bronchoesophageal fistula, *TEF* tracheoesophageal fistula, *T-BEF* tracheobronchial esophageal fistula, *CCRT* Concurrent chemoradiotherapy, *3DCRT* 3-dimensional conformal radiation therapy, *IMRT* Intensity-modulated radiotherapy

^a^Closing of fistula was found at 6 months’ follow-up. She performed Ivor-Lewis operation due to persistent residual mass and pathology showed complete response

**Fig. 4:**
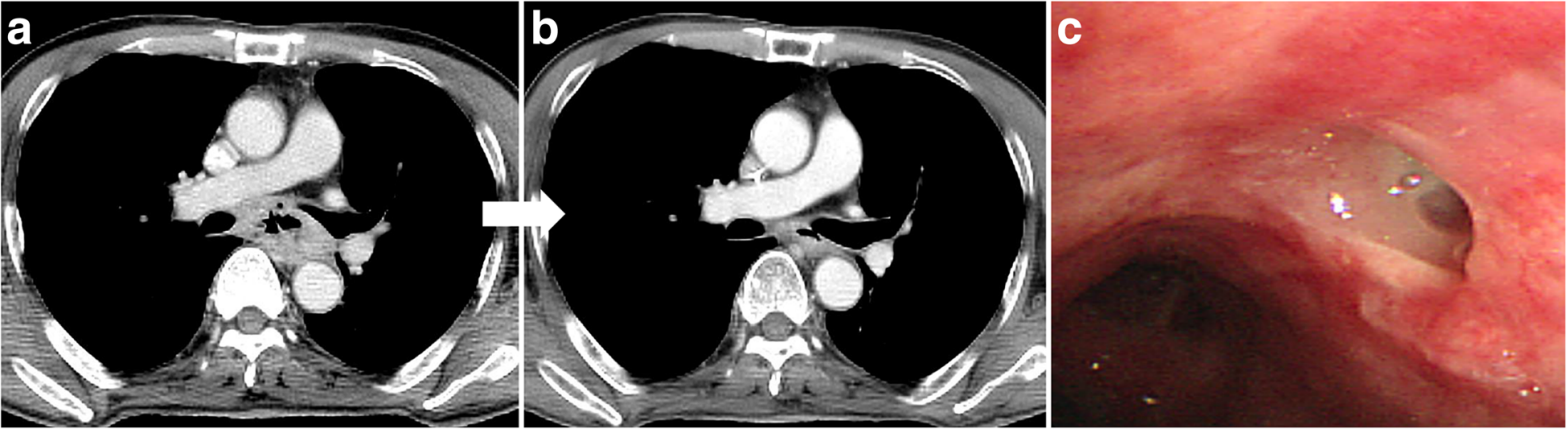
Chest CT and bronchoscopy images at pre-treatment (**a**) and 1-month follow-up (**b** and **c**) in a patient with newly developed bronchoesophageal fistula after treatment

Newly developed oesophageal fistulae after treatment were observed in 13 patients (27.7%, Fig. 4). The median time from treatment initiation to fistula diagnosis was 3 months (range, 1–15). The clinical courses of patients with oesophageal fistulae newly developed after treatment are summarized in Table 3. The fistula closed in one of the 13 patients during the follow-up; he could eat a normal regular diet at the last follow-up date. Nine patients died from aspiration pneumonia. Despite the lack of statistical significance due to the limited number of cases (*p* = 0.770), disease with gross invasion on bronchoscopy showed a higher incidence of fistula development than did disease with mucosal change (37.5% vs. 25.0%, respectively). In addition, a higher dose was not associated with a higher risk of fistula formation (*p =* 0.141). Patients treated with < 60 Gy (9/21 patients, 42.9%) showed a higher risk of developing fistula compared to that in patients treated with ≥60 Gy (4/19, 21.1%).

**Table 3:** Clinical courses of patients with esophageal fistula newly developed after treatment

No.	Age/Sex	PS	Treatment	Time to fistula formation	Site	Management	Diet at last f/u	Survival (month)	Status
1	60/M	1	Definitive CCRT, 62Gy/34Fx	6 months	TEF	Feeding- jejunostomy	Tube feeding	8	DOD (aspiration pneumonia)
2	65/M	1	Definitive RT alone, 54Gy/18Fx	1 month	TEF	PEG	Tube feeding	2	DOD (aspiration pneumonia)
3	68/M	1	Neoadjuvant CCRT, 45Gy/25Fx	15 months	TEF	None	None	15	DOD (aspiration pneumonia, bleeding)
4	68/M	2	Palliative RT, 30Gy/10Fx	1 month	BEF	PEG	Tube feeding	3	DOD (aspiration pneumonia)
5	54/M	1	Palliative RT, 30Gy/12Fx	3 months	BEF	PEG	Tube feeding	4	DOD (aspiration pneumonia)
6	74/M	2	Definitive RT alone, 60Gy/20Fx	3 months	TMF	PRG	Tube feeding	4	DOD (disease progression)
7	65/M	2	Palliative RT, 30Gy/10Fx	3 months	TEF	Feeding- jejunostomy	Tube feeding	7	DOD (aspiration pneumonia)
8	63/M	1	Neoadjuvant CCRT, 44Gy/22Fx	1 month	BEF	Feeding- jejunostomy	Oral intake	29	Alive with no evidence of disease^a^
9	61/M	3	Palliative RT, 30Gy/10Fx	1 month	TEF	PEG	Tube feeding	4	DOD (aspiration pneumonia)
10	48/M	1	Palliative RT, 39Gy/13Fx	After ICT	TEF	PEG	Tube feeding	6	DOD (aspiration pneumonia)
11	70/M	2	Palliative RT, 39Gy/13Fx	After ICT	BEF	PEG	Tube feeding	4	DOD (aspiration pneumonia)
12	56/M	1	Definitive CCRT, 66Gy/33Fx	8 months	TEF	PRG	Tube feeding	12	Alive with no evidence of disease
13	59/M	1	Definitive CCRT, 66Gy/33Fx	5 months	TEF	PRG	Tube feeding	11	Alive with disease

*Abbreviation: PS* performance status, *RT* radiotherapy, *CCRT* Concurrent chemoradiotherapy, *BEF* bronchoesophageal fistula, *TEF* tracheoesophageal fistula, *PEG* percutaneous endoscopic gastrostomy, *PRG* percutaneous radiological gastrostomy, *DOD* dead of disease

^a^BEF was newly developed at 1 month’ follow-up after completion of RT. Later, closing of fistula was found at 2 months’ follow-up and he performed operation and still alive with no evidence of disease

Severe oesophageal bleeding causing death occurred in two patients, including one who developed a fistula at once and died immediately after diagnosis. There was no interruption in RT except for two patients who had jejunostomy wound infection and cardiac event during RT, respectively.

## Discussion

In patients with oesophageal cancer, airway invasion with or without a fistula presents a challenge for therapy. In previous studies, the use of CCRT demonstrated long-term survival and closing of fistulae in a minority of patients. The first study from Japan [10] showed promising results of CCRT for patients with an oesophageal fistula. Closing of the fistula was observed in 71% of patients, with an MS time of 6.6 months from the fistula diagnosis. Later, the use of 60 Gy CCRT in 30 fractions with a protracted infusion of 5-FU and cisplatin for clinical T4 oesophageal cancer including both T4a and T4b was performed [11]. For patients with stage III disease, the MS time and 2-year OS rate were 12 months and 27%, respectively. Two of five clinical T4b tumours with fistulae showed disappearance of the fistula after RT. However, the aggravation or development of a fistula was noted in five of 25 patients (20%). Another study reported the results of CCRT in patients with a fistula that developed before or during treatment [12]. The MS time was 8.5 months and the 1- and 2-year OS rates were 33 and 22%, respectively. Disappearance of the fistula was noted during or after CCRT in seven of 16 patients (44%). However, treatment was terminated early for five patients (31%) because of worsening of the oesophageal fistula, including two treatment-related deaths (13%).

In the current study, the MS time for patients treated with a curative aim was 15 months and the 1- and 2-year OS rates were 51.8 and 34.3%, respectively. A significantly increased survival was achieved in patients treated with a curative aim compared to that in patients treated with a palliative aim (*p* = 0.001). In addition, the CCRT group showed a better survival than that in the RT-only group (*p* = 0.009). Like previous studies, closing of an oesophageal fistula was observed in about half of patients with fistula before treatment (3/7, 42.9%). Fistula aggravation and newly developed fistula were observed in one patient and 13 patients, respectively. Interestingly, only one patient (1/13, 7.7%) later experienced fistula closure. Nine of 13 patients died from aspiration pneumonia. Oesophageal fistulae developed after treatment might be associated with a lower chance for closing and a higher risk for aspiration pneumonia compared to those for a fistula present before treatment.

We tried to identify risk factors for fistula development. In a previous study [13, 14], oesophageal stenosis, circumferential involvement, and elevated C-reactive protein level were associated with the risk of fistula. In the current study, disease with gross invasion on bronchoscopy showed a higher incidence of fistula development than that for disease with mucosal change (37.5% vs. 25.0%, respectively), although the difference was not statistically significant (*p* = 0.770). The RT dose was also not associated with the risk of fistula.

As shown above, severe complications, including fistula development and treatment-related death, have been a major challenge in the treatment of patients with clinical T4b disease with airway invasion. Thus, a strategy to reduce these severe complications is needed. Several studies demonstrated several benefits of induction chemotherapy (ICT) as an initial treatment for patients with clinical T4 disease [15, 16]. This treatment might effectively downstage the tumour, improve the resectability, anticipate treatment response, and lower the rate of fistula formation. In 2007, the results of a phase II trial of docetaxel, cisplatin, and 5-FU (DCF) followed by CCRT in patients with unresectable, locally advanced oesophageal squamous-cell carcinoma were released. In a case report of oesophageal cancer with airway invasion treated with ICT followed by CCRT [17], the planned total dose was 50.4 Gy with concurrent cisplatin and irinotecan. There was no evidence of disease until the last follow-up date. Later, the efficacy of ICT using DCF for clinical T4 oesophageal squamous-cell carcinoma was evaluated [18]. Fifty patients who underwent ICT using DCF were propensity-score-matched with 50 patients who underwent CCRT with 5-FU and cisplatin. The DCF group had significantly higher overall resectability compared to that in the CCRT group (78.0% vs. 48.0%, *p* < 0.01). The oesophageal perforation rate during induction treatments was significantly lower in the DCF group than that in the CRT group (4.0% vs. 18.0%, *p* = 0.02). The prognosis was significantly better in the DCF group than that in the CRT group (5-year cancer-specific survival 42.1% vs. 22.2%, *p* = 0.01). Against this, we experienced fistulae aggravation in two of four patients after ICT with a regimen of 5-FU and cisplatin. These two patients were treated with palliative RT and all died from aspiration pneumonia during follow-up. One patient had a successful treatment with ICT followed by RT. The patient, who had a gross left main bronchus invasion of oesophageal cancer, was treated with four cycles of docetaxel and cisplatin followed by sequential RT with a total dose of 50 Gy in 20 fractions. During follow-up, there was no evidence of fistula development or disease progression until the last follow-up date. To determine the appropriate strategy for ICT, further studies on effective chemotherapeutic regimens are needed.

Our current study had several limitations. First, it was a retrospective study; therefore, some selection bias was possible. Second, there might have been chronological changes in radiotherapy techniques during the study period.

## Conclusions

Even for clinical T4b disease with airway invasion, radiation therapy with a curative aim showed acceptable survival outcomes in patients with good performance status and no distant metastasis at initial diagnosis. However, the risk of fistula development associated with fatal events remains high. Further study on the association between ICT with an effective regimen and the risk of fistula development is warranted to decrease the risk of treatment and improve clinical outcomes.

## Abbreviations

3D-CRT: Three-dimensional conformal RT
AJCC: American Joint Committee on Cancer
CCRT: Concurrent chemoradiotherapy
CT: Computed tomography
CTV: Clinical target volume
DCF: Docetaxel, cisplatin, and 5-FU
EGD: Esophagogastroduodenoscopy
FDG-PET-CT: 18F-fluorodeoxyglucose positron-emission tomography with CT
GTV: Gross tumour volume
ICT: Induction chemotherapy
IMRT: Intensity-modulated radiation therapy
MS: Median survival
NCCN: National Comprehensive Cancer Network
OS: Overall survival
PTV: Planning target volume
RT: Radiation therapy
